# Dissecting the fission yeast regulatory network reveals phase-specific control elements of its cell cycle

**DOI:** 10.1186/1752-0509-3-93

**Published:** 2009-09-16

**Authors:** Pierre R Bushel, Nicholas A Heard, Roee Gutman, Liwen Liu, Shyamal D Peddada, Saumyadipta Pyne

**Affiliations:** 1 Biostatistics Branch, National Institute of Environmental Health Sciences, Research Triangle Park, NC 27709 USA; 2 Department of Mathematics, Imperial College, London, UK; 3 Department of Statistics, Harvard University, Cambridge, MA 02138, USA; 4 Broad Institute of the Massachusetts Institute of Technology and Harvard University, 7 Cambridge Center, Cambridge, MA 02142, USA; 5 Department of Statistics and Department of Environmental Health, Harvard School of Public Health, Harvard University, Boston, MA 02115, USA; 6 Department of Medical Oncology, Dana-Farber Cancer Institute, Harvard Medical School, Boston, MA 02115, USA

## Abstract

**Background:**

Fission yeast *Schizosaccharomyces pombe *and budding yeast *Saccharomyces cerevisiae *are among the original model organisms in the study of the cell-division cycle. Unlike budding yeast, no large-scale regulatory network has been constructed for fission yeast. It has only been partially characterized. As a result, important regulatory cascades in budding yeast have no known or complete counterpart in fission yeast.

**Results:**

By integrating genome-wide data from multiple time course cell cycle microarray experiments we reconstructed a gene regulatory network. Based on the network, we discovered in addition to previously known regulatory hubs in M phase, a new putative regulatory hub in the form of the HMG box transcription factor *SPBC19G7.04*. Further, we inferred periodic activities of several less known transcription factors over the course of the cell cycle, identified over 500 putative regulatory targets and detected many new phase-specific and conserved *cis*-regulatory motifs. In particular, we show that *SPBC19G7.04 *has highly significant periodic activity that peaks in early M phase, which is coordinated with the late G2 activity of the forkhead transcription factor *fkh2*. Finally, using an enhanced Bayesian algorithm to co-cluster the expression data, we obtained 31 clusters of co-regulated genes 1) which constitute regulatory modules from different phases of the cell cycle, 2) whose phase order is coherent across the 10 time course experiments, and 3) which lead to identification of phase-specific control elements at both the transcriptional and post-transcriptional levels in *S. pombe*. In particular, the ribosome biogenesis clusters expressed in G2 phase reveal new, highly conserved RNA motifs.

**Conclusion:**

Using a systems-level analysis of the phase-specific nature of the *S. pombe *cell cycle gene regulation, we have provided new testable evidence for post-transcriptional regulation in the G2 phase of the fission yeast cell cycle. Based on this comprehensive gene regulatory network, we demonstrated how one can generate and investigate plausible hypotheses on fission yeast cell cycle regulation which can potentially be explored experimentally.

## Background

Fission yeast *Schizosaccharomyces pombe *and budding yeast *Saccharomyces cerevisiae *are among the original model organisms in the study of the cell-division cycle [1]. In particular, our understanding of the cell cycle of *S. cerevisiae *was greatly enhanced over the last decade owing to several genome-wide expression studies [2]. Based on such high-throughput studies, detailed regulatory networks for budding yeast were reconstructed on genome-wide scale and complexity, e.g., [3,4]. Yet, unlike *S. cerevisiae*, no comprehensive cell cycle regulatory network is known for *S. pombe*. It has only been partially characterized [2]. As a result, important regulatory cascades in budding yeast have no known or complete counterpart in fission yeast.

Past comparisons of networks, which revealed both conserved and divergent control elements for similar modules across different yeasts species [5], highlighted the importance for constructing a large-scale regulatory network for fission yeast. Recently, three independent microarray studies have generated genome-wide time course gene expression data for the cell cycle of *S. pombe *[6-8] making it currently the organism with the largest cell cycle transcriptome. A subsequent meta-analysis [9] of ten experiments from the three studies was a first step towards aligning the data sets but no major attempt to reconstruct a *S. pombe *regulatory network has been made. Using one of the gene expression time course studies of the *S. pombe *cell cycle, Nachman and Regev [10] used a Biochemical Regulatory Network Inference (BRNI) method to identify transcriptional- and motif-based modules comprising some of the known and novel regulatory networks that control the fission yeast cell cycle. However, the limited gene expression data set representing a single form of cell cycle synchronization (elutriation), the noise inherent in the small data set and phase-specific differences between genes and transcription factors (TFs) all pose real challenges to the discovery of a comprehensive and reliable regulatory network.

In this study, we reconstructed a global gene regulatory network along with a comprehensive parts-list of co-regulated genes and significant regulators to allow system-level modularization of the interconnected processes involved in the *S. pombe *cell cycle. We adopted two algorithmic strategies for constructing the parts-list: 1) using the estimates of time period and phase angles from a non-linear model, we computed phase-specific time-lagged correlations between TF-gene pairs to infer the activities of a large set of regulators and 2) partitioning a large pool of genes into clusters that are not only co-expressed in many of the ten time course experiments but are also co-regulated in the ten TF deletion and over expression experiments. These two workflows converged to produce a phase-specific network of regulatory modules. Finally we dissected the network to identify *cis*-elements that may indicate putative mechanisms for phase-specific transcriptional and post-transcriptional regulation of the fission yeast cell cycle.

For the researcher, the modularization of the *S. pombe *cell cycle by our gene and module networks allows for investigation of various phase-specific hypotheses on fission yeast cell cycle regulation. Due to the lack of strong evidence of transcriptional regulation in the G2 phase of the *S. pombe *cell cycle, as compared with *S. cerevisiae*, it was speculated that post-transcriptional regulation might be more active in fission yeast [2]. Indeed among the enhanced clusters of ribosome biogenesis genes that express during the G2 phase in *S. pombe*, we identified new, highly significant and distinctive RNA motifs in their 3' UTR sequences, thus lending support to that hypothesis. We provided evidence for transcript decay of these genes with the help of statistical analysis of their time course profiles and data from previous experiments. Thereby, we also demonstrated how our parts-list could be systematically mined for generating interesting experimentally testable hypotheses.

## Results

### New modules and regulators at M and G1 phases

**Figure 1:**
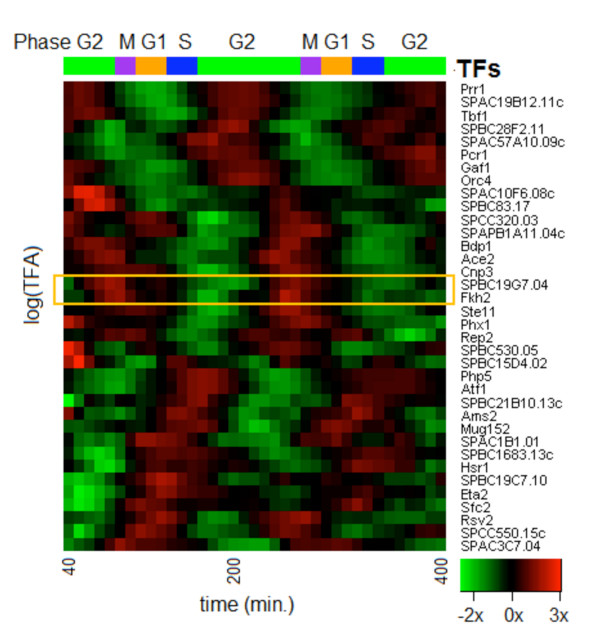
**Transcription factor activities over the course of cell cycle**. Inferred activities of the 36 selected TFs, clustered with respect to similarity of activities based on Peng Cdc25. The cell cycle phases are indicated. TFs *fkh2 *and *SPBC19G7.04 *are highlighted with a rectangle.

**Figure 2:**
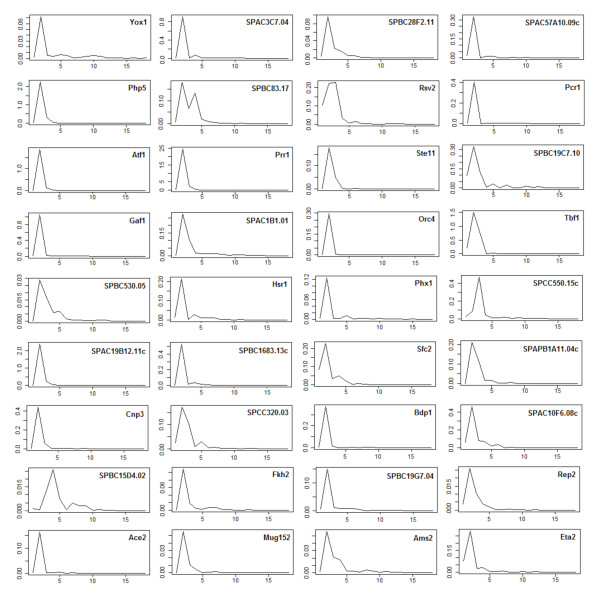
**Statistical significance of the transcription factor activities**. Most of the strongly periodic activity profiles (log_10_(TFA)) of the 36 selected TFs have a single dominant frequency of oscillation. The x-axis marks the different Fourier frequencies and the y-axis represents the Average Periodogram [13]. Clearly the dominant Fourier frequency, marked by a spike, is very prominent in every TF, and is indicative of its strongly periodic profile. Further, the similarity among the locations of the spikes across all 36 TFs shows their regulation by common cell cycle processes. Also see Table 1 for the related p-values that measure the periodicity of each profile.

Using a curated collection of 125 TFs in *S. pombe *with known or predicted protein domains [11], we computed phase-specific time-lagged correlation of expression profiles of all TF-gene pairs. The statistically significant (within each experiment) and consistent (across multiple experiments) correlations helped us identify a TF that is active within a phase-specific regulatory context as well as its targets therein. Specifically, a TF's activity (or TFA) is its weighted average effect as a regulator on the downstream targets at a given time point. Using Network Component Analysis (NCA) [12] we inferred strongly periodic TFAs for 36 TFs (listed in Table 1) with respect to 531 target genes (Additional file 1) during different phases of the cell cycle (Figure 1). Indeed the activity profile (Additional file 2) for almost every TF (in the form log_10 _(TFA) time courses) had a single, dominant periodic component as determined by the Average Periodogram method and the p-value from the related *g*-statistic [13] (Table 1, Figure 2). The Average Periodogram for each of the time courses plotted in Figure 2 is used to detect the presence of a dominant frequency of cell cycle oscillation. Almost every TF shows a single principal oscillation frequency in mutual agreement with the one cycle period. This is indicative of their regulation by common regulatory processes in a strongly periodic manner. While we identified most of the TFs that are GO annotated for cell cycle regulation, many new candidates were also inferred (e.g. an early M phase HMG box TF *SPBC19G7.04*).

**Table 1:** Details about the 36 transcription factors with significant inferred activities.

**Cluster #**	**TF coding gene**	**Periodic Activity P-value**	**Protein domain**
	Prr1	5.70E-15	HSF-type DNA-binding

26	SPAC19B12.11c	1.77E-15	Zinc finger, C2H2 type

22	Tbf1	2.27E-06	Myb-like DNA-binding domain

7	SPBC28F2.11	8.20E-07	HMG (high mobility group) box

21	SPAC57A10.09c	2.86E-12	HMG (high mobility group) box

12	Pcr1*	3.83E-26	Basic region leucine zipper/bZIP

17	Gaf1	3.91E-23	GATA zinc finger

22	Orc4	1.85E-25	HMG-I and HMG-Y, DNA-binding/AT-hook

	SPAC10F6.08c	2.66E-06	HMG (high mobility group) box

	SPBC83.17	1.75E-03	Helix-turn-helix

14	SPCC320.03	1.96E-04	Fungal Zn(2)-Cys(6) binuclear cluster domain

	SPAPB1A11.04c	1.35E-05	Fungal Zn(2)-Cys(6) binuclear cluster domain

4	Bdp1	4.27E-16	Myb-like DNA-binding domain

30	Ace2*	3.23E-16	Zinc finger, C2H2 type

11	Cnp3	2.04E-14	HMG-I and HMG-Y, DNA-binding/AT-hook

31	SPBC19G7.04	3.06E-10	HMG (high mobility group) box

31	Fkh2*	7.76E-09	Fork head domain

	Ste11	4.97E-10	HMG (high mobility group) box

23	Phx1	1.04E-08	Homeobox domain

7	Rep2*	1.96E-05	Zinc finger, C2H2 type

22	SPBC530.05	6.90E-04	Fungal Zn(2)-Cys(6) binuclear cluster domain

	SPBC15D4.02*	1.82E-03	Fungal Zn(2)-Cys(6) binuclear cluster domain

21	Php5	4.11E-14	Histone-like TF (CBF/NF-Y)

12	Atf1*	9.07E-17	Basic region leucine zipper/bZIP

	SPBC21B10.13c	3.34E-07	Homeobox domain

31	Ams2*	1.41E-04	GATA zinc finger

5	Mug152	2.08E-09	Myb-like DNA-binding domain

22	SPAC1B1.01	5.28E-06	Zinc finger, C2H2 type

11	SPBC1683.13c	1.17E-13	Fungal Zn(2)-Cys(6) binuclear cluster domain

16	Hsr1	1.62E-08	Zinc finger, C2H2 type

	SPBC19C7.10	1.55E-04	APSES domain

9	Eta2	3.29E-07	Myb-like DNA-binding domain

24	Sfc2	4.47E-05	Zinc finger, C2H2 type

12	Rsv2	6.46E-03	Zinc finger, C2H2 type

26	SPCC550.15c	3.60E-08	Zinc finger, C2H2 type

	SPAC3C7.04	2.84E-15	Fungal Zn(2)-Cys(6) binuclear cluster domain

*Denotes a TF that is annotated as cell cycle relevant in Gene Ontology (GO).

**Figure 3:**
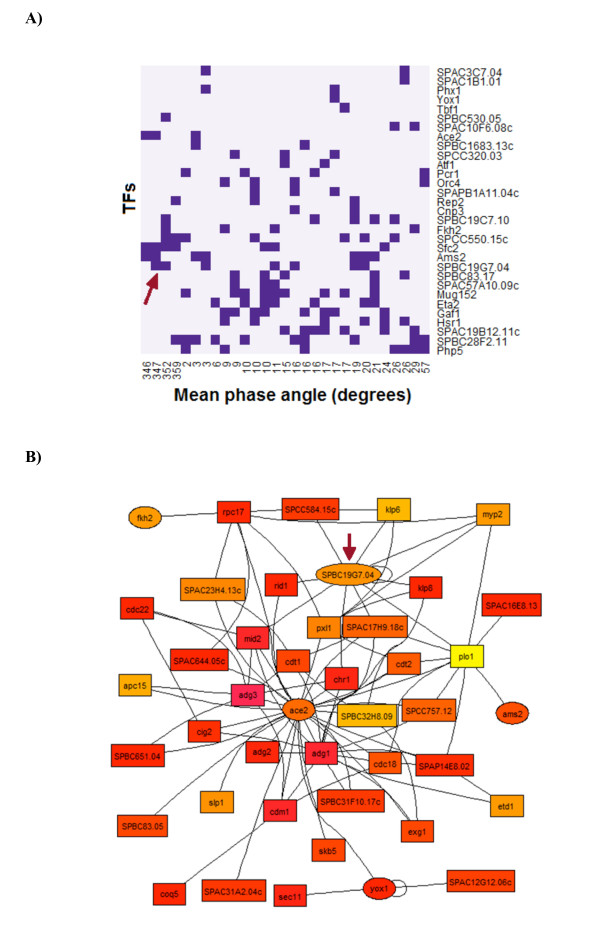
**Dissecting M and G1 phase subnetworks. A**) A network of M and G1 phase gene modules are shown, sorted by mean phase. If a TF is found to be a regulator for a module then it is depicted with a purple square. Potential regulation of M phase modules by *SPBC19G7.04 *is pointed out with an arrow. **B**) Early M phase gene regulatory network in which TFs are shown in ellipses, and the rest of the genes in boxes. The colors of the nodes are according to their peak phase, where more reddish colors represent phases that are later in M and thereafter, while more orange and yellow hues depict earlier (late G2 and G2/M) phases. The regulatory hub for the early M phase TF *SPBC19G7.04 *is marked with an arrow.

Based on the regulatory matrix of the 36 TFs and their downstream targets in multiple experiments, we reconstructed a gene regulatory network (Additional file 3), which could be mined for inferring phase-specific modules and sub-networks. For instance, a majority of the 36 TFs were found to regulate one or more modules in the M and G1 phases (Figure 3A and see Additional file 4). In addition, specific gene-level regulatory links may be studied by dissecting the global network. For instance, a subnetwork of early M phase clusters (Figure 3B) revealed a new regulatory hub in the form of the HMG box TF *SPBC19G7.04 *(denoted by an arrow in Figure 3B), besides such well-known hubs as the key TF *ace2 *and the polo kinase *plo1*.

### Clusters of co-regulated genes lead to new phase-specific control elements

**Figure 4:**
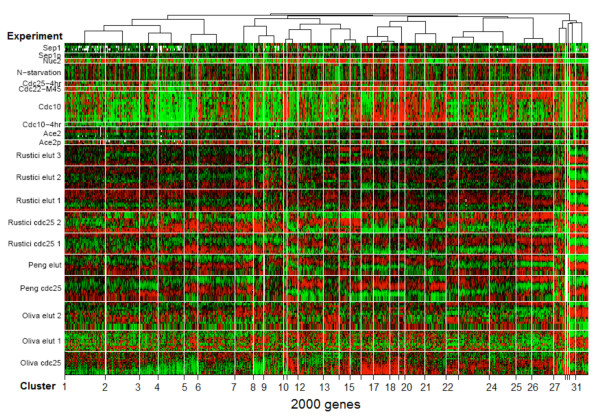
**Co-expressed gene clusters with regulatory signatures**. Data from ten cell cycle time course experiments were integrated with ten regulatory TF knockout and overexpression experiments to produce 31 clusters. Columns are clusters of 2000 fission yeast genes and each row an experiment. One cycle long time courses are shown depicting the cyclic (high-and-low) expression for every cluster. (Color code: red-high, green-low, white-missing expression.)

**Figure 5:**
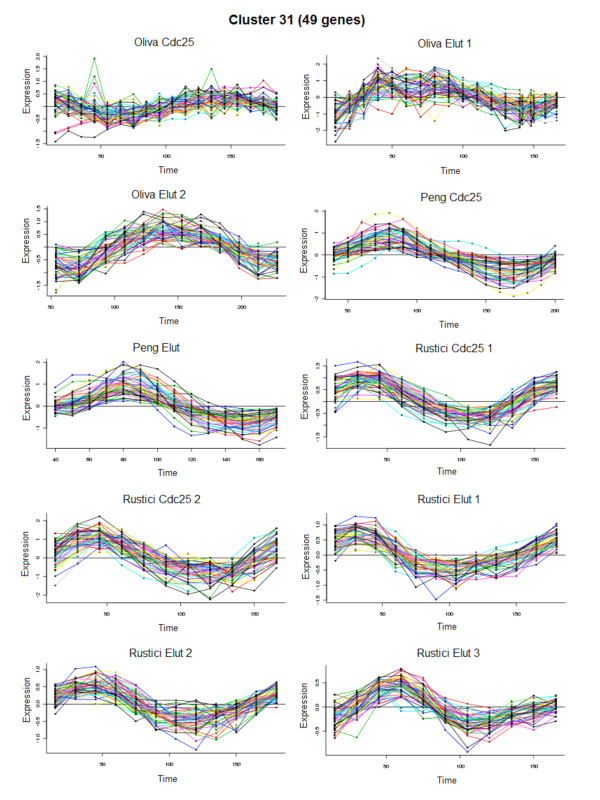
**A representative co-expressed gene cluster identified with the revised co-clustering algorithm**. Cell cycle time courses of 49 genes belonging to cluster 31 in mid-M phase are shown.

As a basis of our large-scale regulatory network, we sought out to construct a parts-list (as defined in [14]) of statistically significant cell cycle components such as a large number of co-regulated gene clusters and their putative regulators. To increase statistical power for detection of new or subtle modes of regulation, we enhanced a Bayesian algorithm [15] with new strategies to co-cluster different types of experiments and identify co-regulated genes. The revised algorithm accommodates heterogeneous data types by modeling (individually) each of the ten cell cycle time course experiments and the ten TF deletion or overexpression experiments with suitable basis functions. With the help of effective priors to model the periodically expressed profiles apart from the monotonic trends, the algorithm produced an optimal clustering of 2000 fission yeast genes into 31 disjoint clusters (Figure 4, and Additional file 5 Figure S1). Details regarding the 31 clusters of co-expressed genes are summarized in Table 2. A complete list of the 2000 genes categorized by the cluster numbers is in Additional file 6. We also determined the regulatory signature of every cluster (Additional file 5 Figure S2). An example of a periodic cluster containing 49 genes expressed in M phase is shown in Figure 5.

**Table 2:** Details about the 31 clusters of co-expressed genes.

**Cluster #**	**Size**	**Mean phase (degrees)**	**p-value for phase uniformity**	**Significant motif**	**Dominant Gene Ontology category**
9	76	6	4.54E-01		transmembrane transporter activity

5	52	17	7.81E-08		cellular metabolic process, cellular component organization and biogenesis

14	42	42	6.41E-04	Mcb1	nucleotide binding, substrate-specific transporter activity

21	80	49	2.07E-17	G.TTGT [TG] [AG]	oxidoreductase activity

29	9	53	6.84E-12	Histone CACCACC	histones

15	41	55	1.49E-18	GTTGGC [AT]GT	ion binding

10	12	95	3.54E-02		cellular metabolic process, protein binding, stress response

12	96	102	3.52E-04		transmembrane transporter activity

20	78	103	1.27E-07	CAAGTT	transport, establishment of localization

18	34	108	1.89E-16		cellular metabolic process, nitrogen compound metabolic process

17	61	112	4.52E-12		cellular metabolic process, cofactor binding

16	49	135	1.57E-25	GTT.GCT	nitrogen compound metabolic process

27	45	139	1.76E-02		transmembrane transporter activity

28	8	150	8.00E-07		

19	24	174	9.65E-15	GTGACTG [CT]T TAGGGTAGGG	cellular metabolic process, structural constituent of ribosome

22	47	175	5.38E-02	CGTTAGTTTT	regulation of metabolic process

23	118	175	1.03E-06		

26	83	188	1.90E-39	AGTTTG	cellular component organization and biogenesis

11	43	194	9.52E-04	GTCGGTTTCC	transmembrane transporter activity

24	103	195	3.52E-18		cellular metabolic process, ion binding

25	59	211	4.09E-02	[GT]TAAACA	ribosome biogenesis and assembly

3	70	255	1.06E-07	TGTTTAC	cell cycle, chromosome segregation

13	60	258	9.74E-05		cell cycle

1	155	259	2.21E-11	FKH	cell cycle, stress response, cellular metabolic process, protein binding, response to endogenous stimulus

2	131	276	3.34E-13	FKH TCTTCT	cell cycle

4	101	286	1.07E-06	FKH TGTAAGC	cell cycle, cellular component organization and biogenesis

8	41	328	1.74E-02	ACCATTG FKH	primary metabolic process, ion binding, regulation of biological quality

6	140	343	1.39E-03	T [GC]GTG [TG]T	cellular component organization and biogenesis

31	49	344	1.47E-33	FKH	cell division, cytokinetic process

30	22	357	6.56E-05	Dbl10 Ace2	cell cycle, regulation of metabolic process

7	71	359	6.15E-13	Ace2	cell cycle, cell communication

Motifs similar to known motifs are given by the name published in the literature (ACCACA ~ Mcb1, CCCTTACCC ~ Histone, TTGTTTAC ~ FKH, CGTGTCGCGT ~ Dbl10, ACCAGCC ~ Ace2). Newly discovered motifs are given by their consensus sequence.

The co-regulated genes within the clusters were characterized and validated. Gene Ontology (GO) analysis revealed that many of the genes within the clusters were represented by known functional categories from different stages of the cell cycle (Table 2). Testing of circular uniformity of peak phase angles of genes in each cluster determined that 29 out of 31 clusters were cell cycle phase-specific. Circular-circular Regression (CCR) [16] showed that during cell cycle progression, the phase ordering of the 31 clusters exhibited significant (*P *= 0.037) coherence across the ten time course experiments. Significant (*P *< 10^-9^) and non-redundant putative binding sites, many of which were conserved across different fission yeasts, were detected for most of the clusters (Table 2). Several new and interesting motifs were observed (e.g. TGTAWGC in Cluster 4) beside some that were previously known (e.g. the forkhead FKH motif TTGTTTAC).

### Post-transcriptional regulation of ribosome biogenesis genes

**Figure 6:**
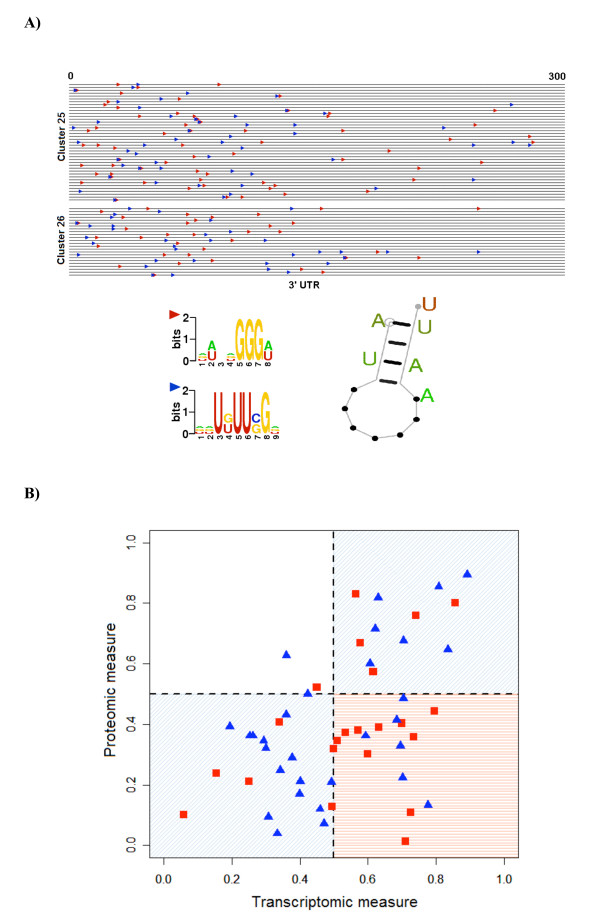
**Post-transcriptional regulation of ribosome biogenesis. A**) Genes from the clusters for ribosome biogenesis and related protein assembly and cellular component organization containing statistically significant and conserved RNA motifs in their 3' UTR first 300 bases. The two motifs (see logos) have positional and directional bias and sometimes appear as a combined motif. The genes are listed in the displayed ordering in Table 3. The predicted structure of the motif with the highest likelihood score is also shown. **B**) Post-transcriptional *cis*-regulation at these motifs is tested against previously published experimental data [21]. The red points mark the transcriptomic versus proteomic levels of the genes with the motifs while the blue points depict that for genes from the same clusters without the motifs.

Post-transcriptional regulation plays a key role in the control of gene expression in terms of processing, transport, localization, quality control and turnover of mRNA transcripts. Therefore, systematic identification of targets for such regulation is of fundamental importance to the investigation of multi-layered gene regulation [14,15]. In the present study, we identified new, highly conserved motifs in the 3' UTR sequences of 65 co-regulated genes from clusters 25 and 26 that are involved mostly in ribosome biogenesis in *S. pombe *(Figure 6A; the genes are listed in Table 3). Two single-stranded motifs U [UG]UU [CG]G and GGG [AU] in 3' UTR [17] were highly statistically significant (*P *< 10^-47 ^and 10^-67 ^respectively) with strong positional bias within the range of the first 300 nucleotides. In the most significant occurrence of the resulting RNA motif (as in *SPBC26H8.08c*), they appeared overlapped as UUCUUCGGGUUUUAA with a small loop structure, denoted by (see Figure 6A). See Additional file 7 for associated likelihood scores. Dominant GO categories of biological process and cellular component for the proteins encoded by the genes containing the motifs include RNA processing (*P *< 10^-39^) and nucleus-nucleolus (*P *< 10^-52^) respectively (the gene products are described in Table 3).

**Table 3:** Genes from clusters 25 and 26 carrying the 3'UTR RNA motifs (the list is ordered exactly as depicted in Figure 6A).

**Gene**	**Cluster**	**Gene Product**	**P-value of expression**	**Skew**	**Kurtosis**
SPAC1565.05	25	Ribosomal protein L29	2.90E-03	-0.348	-1.555

SPAC16E8.06c	25	RNA-binding protein Nop12	3.89E-04	-0.524	-0.532

SPAC18B11.06	25	U3 snoRNP-associated protein Lcp5	1.18E-05	0.315	-0.893

SPAC222.06	25	nuclear HMG-like acidic protein Mak16	2.29E-05	0.827	-0.185

SPAC22E12.13c	25	60S ribosomal protein L24-3 (L30)	7.69E-05	-0.314	-1.715

SPAC22E12.18	25	Uncharacterized protein C22E12.18	2.99E-03	-0.256	-1.451

SPAC22F3.08c	25	ATP-dependent RNA helicase Rok1	2.26E-02	0.747	0.049

SPAC22F8.09	25	rRNA processing protein Rrp16	3.16E-04	0.509	0.150

SPAC26A3.06	25	methyltransferase	7.17E-03	0.237	-1.379

SPAC2E1P5.05	25	U3 snoRNP-associated protein Rrp9	9.06E-04	-0.554	-1.587

SPAC3G9.15c	25	rRNA processing protein Fcf2	1.15E-02	-0.064	-1.423

SPAC4F8.04	25	Brix domain protein Rpf1	2.64E-03	0.028	-0.944

SPAC56F8.09	25	rRNA methyltransferase Rrp8	1.91E-04	-0.299	-1.422

SPAC57A7.06	25	U3 snoRNP protein Utp14	6.01E-05	0.174	-1.597

SPAC664.08c	25	traub family protein	1.52E-07	-0.239	-1.455

SPAC683.02c	25	zf-CCHC type zinc finger protein	1.10E-06	0.686	0.119

SPAC823.08c	25	ATP-dependent RNA helicase Rrp3	1.51E-03	-0.050	-1.231

SPAC890.05	25	ribosome biogenesis protein	1.17E-05	0.482	-0.181

SPAC926.08c	25	Brix domain protein Rpf2	6.14E-08	-0.114	-1.409

SPBC11G11.03	25	ribosome assembly protein	6.15E-04	-0.241	-1.781

SPBC13G1.09	25	bystin-family protein	1.04E-07	0.170	-0.725

SPBC14C8.14c	25	DNA polymerase phi	6.61E-02	-0.290	-1.667

SPBC1604.09c	25	exoribonuclease Rex4	5.43E-04	0.605	0.702

SPBC1711.04	25	methylenetetrahydrofolate reductase	6.71E-01	0.397	-1.564

SPBC1718.03	25	DNA-directed RNA polymerase I complex subunit Ker1	7.68E-06	-0.048	-1.673

SPBC1734.01c	25	pre-rRNA processing protein Esf1	4.48E-02	-0.095	-1.293

SPBC19F5.05c	25	pescadillo-family BRCT domain protein	4.23E-05	-0.448	-1.747

SPBC215.06c	25	human LYHRT homolog	1.53E-05	0.088	-1.519

SPBC24C6.02	25	ATP-dependent RNA helicase Spb4	7.51E-05	0.292	-0.529

SPBC26H8.08c	25	GTPase Grn1	3.79E-05	-0.450	-1.602

SPBC28E12.05	25	U3 snoRNP-associated protein Esf2	2.75E-01	0.511	-0.565

SPBC2D10.19c	25	pre-60S shuttlingfactor	1.23E-04	-0.268	-1.346

SPBC2G5.03	25	cytosolic thiouridylase subunit Ctu1	6.83E-05	0.770	-0.344

SPBC31E1.06	25	GTP binding protein Bms1	5.75E-04	0.206	-0.677

SPBC336.02	25	18S rRNA dimethylase	2.47E-03		

SPBC409.15	25	rRNA processing protein Tsr2	2.51E-02	-0.071	-1.343

SPBP8B7.10c	25	U3 snoRNP-associated protein Utp16	5.91E-02	-0.215	-1.578

SPCC18.12c	25	rRNA processing protein	4.43E-03	0.305	-1.143

SPCC24B10.18	25	human Leydig cell tumor 10 kDa protein homolog	3.23E-04	0.539	-0.725

SPCC550.15c	25	ribosome biogenesis protein	1.70E-02	-0.495	-1.676

SPCP1E11.11	25	Puf family RNA-binding protein	3.26E-04	-0.565	-1.238

SPAC1527.03	26	RNA-binding protein	2.14E-04	-0.239	-1.786

SPAC15A10.04c	26	EF-1 alpha binding zinc finger protein Zpr1	1.02E-04	-0.336	-1.436

SPAC1687.11	26	rRNA methyltransferase Spb1	1.06E-04	0.275	-1.080

SPAC23H4.15	26	ribosome biogenesis protein Tsr1	4.81E-04	-0.333	-1.788

SPAC30C2.02	26	deoxyhypusine hydroxylase	3.06E-04	-0.347	-1.621

SPAC31A2.07c	26	ATP-dependent RNA helicase Dbp10	5.04E-02	-0.138	-1.519

SPAC4F8.12c	26	U5 snRNP complex subunit Spp42	6.80E-01	-0.440	-1.721

SPAC6F12.16c	26	ATP-dependent RNA helicase, TRAMP complex subunit Mtr4	2.66E-04	-0.266	-1.411

SPAPB1A10.06c	26	ATP-dependent RNA helicase Dhr1	1.29E-05	-0.048	-1.539

SPBC16E9.10c	26	AAA family ATPase Rix7	6.65E-04	-0.258	-1.735

SPBC16H5.08c	26	ribosome biogenesis ATPase, Arb family ABCF2-like	1.59E-02	-0.044	-1.634

SPBC17D1.06	26	ATP-dependent RNA helicase Dbp3	1.36E-06		

SPBC244.02c	26	U3 snoRNP-associated protein Utp6	2.54E-03	-0.368	-1.599

SPBC4C3.05c	26	DNA-directed RNA polymerase I complex large subunit Nuc1	4.79E-04	-0.103	-1.079

SPBC4F6.07c	26	ATP-dependent RNA helicase Mak5	2.21E-02	0.191	-1.272

SPBC4F6.13c	26	WD repeat/BOP1NT protein	1.11E-03	-0.526	-1.413

SPBC651.01c	26	GTP binding protein Nog1	4.48E-09		

SPBC776.08c	26	Nrap (snoRNA binding)	5.23E-06	-0.424	-1.720

SPBP22H7.02c	26	RNA-binding protein Mrd1	2.43E-06	-0.179	-1.690

SPCC1183.07	26	U3 snoRNP-associated protein Rrp5	9.57E-07	-0.142	-1.475

SPCC1827.01c	26	DUF1253 family protein	1.58E-03	-0.126	-1.760

SPCC320.08	26	membrane transporter	5.16E-05	-0.166	-1.538

SPCC330.09	26	rRNA processing protein Enp2	9.94E-04	0.128	-0.810

SPCC737.08	26	midasin	2.82E-02	-0.309	-1.437

The transcriptome and the proteome have long been compared to gain insights on RNA turnover [18-20]. Thus, to explore the present hypothesis, we analyzed comparative transcriptomic and proteomic measurement data of *S. pombe *gene expression from a previous high-throughput study [21]. Genes with low correlation between their transcriptomic and proteomic levels could be substantially regulated by post-transcriptional mechanisms [22]. Both types of measurements were available in the above data for 22 of the 65 genes that had the motifs, and for 30 of the remaining genes (in clusters 25 and 26) that did not. We first computed the percentile scores separately for each measurement to be able to compare them on the same scale (Figure 6B). As shown in the lower left (blue) quadrant, the few genes with the motifs and low transcript levels are correlated with their protein product expression. Given this data set, perhaps it may be reasonable to assume that the mRNA abundance in the cells is sufficient for carrying out various biological processes during the cell cycle. However, the correlation between the transcriptomic and proteomic levels for the genes with high expression and containing the motifs was lower (*P *= 0.07 not significant for H_0_:*ρ *= 0 at level 0.05) than those without them (significant correlation with *P *= 0.004). Indeed most of the genes carrying the motifs and having above-average transcript level (percentile score > 0.5) have a steadily low protein level (percentile score < 0.5; see red quadrant in Figure 6B) potentially indicative of transcript decay or short half-life. Conversely, the majority of genes without the motifs had a protein level consistent with their transcript level (blue quadrants). Similarly, high correlation (*ρ *= 0.66) was observed for cell cycle genes in general highlighting further the discrepancy between the transcriptomic and proteomic levels of many of the genes that contained the motifs [21]. Given the generally high values of AUG Context Adaptation Index (mean = 0.56, s.d. = 0.15) and ribosome occupancy (mean = 81%, s.d. = 4.3%) of these genes [23], the discrepancy may be more likely due to post-transcriptional regulation than to lack of translational efficiency.

We believe that the shapes of the time-course profiles of genes containing the RNA motifs can provide useful information regarding the pattern of their decay in G2-phase. We used standard statistical measures such as skewness and kurtosis of a time course [24] to describe the decay characteristics. The skew and excess kurtosis statistics measure respectively, the asymmetry and the peakedness of a profile that could shed light on the temporal pattern of its decay. For instance, while a left skewed profile (given by a negative value of skew) may represent an early peaking gene, a heavy right-tailed profile (given by a positive excess kurtosis) denotes slow decay rate. Therefore, we first computed the skew of each of the above 65 profiles restricted to the first full duration of the G2 phase, and then the kurtosis by focusing on the right tail (see Table 3). We noted that most of the genes are expressed in early- to mid-G2 phase, i.e. with left or negative skew, as is consistent with the ribosome biogenesis expression previously observed [6]. Clearly, most profiles had a sharp decline as G2 progresses, which are captured by the negative excess kurtosis of their right tails.

## Discussion

In the study of the systems biology of a unicellular organism such as fission yeast, the importance of the interconnected cell cycle processes cannot be over-emphasized. However, the processes could be studied both in terms of their connectedness to each other as well as their cell cycle phase-specificity. To encompass both the global and the local aspects of the underlying gene regulatory network for *S. pombe*, we took a 3-step approach. First, using genome-wide expression data from multiple experiments, we reconstructed a gene regulatory network based on 531 downstream target genes of 36 transcription factors that were identified to have strongly periodic activity during the cell cycle (Table 1, Figures 1 and 2). Second, we coupled TF mutant data from ten microarray studies with time course expression data from ten cell cycle experiments with the help of an enhanced Bayesian co-clustering algorithm. The co-regulated and phase-specific gene modules (Figure 4) led to the identification of many new conserved *cis*-regulatory elements (Table 2 and Figure 6A). Third, we dissected some parts of the above network to identify cell cycle phase-specific control elements to show how the gene regulatory network and the parts-list could be used for generating hypothesis about *S. pombe *cell cycle regulation.

Using new computational strategies and a large gene pool, we constructed a comprehensive parts-list of key regulatory genes, many interesting TFs and binding motifs, and phase-specific modules that offer insights on different aspects of the fission yeast cell cycle regulatory program (see Figure 6 for example). Beyond a core of 500 strongly cell cycle regulated genes in *S. pombe *[25], based on observed transcript oscillation, it has been noted that the number of genes that might be regulated by the cell cycle, due to reasons that are adaptive or otherwise, could be as many as 2000, approximately two-thirds of which may be weakly regulated [6]. Indeed if we observe the ranking of all genes by the variance of their peak phases across ten independent experiments as determined by Marguerat et al. [9], then the top 2000 genes show surprisingly low variance. Thus, to identify a comprehensive set of co-regulated genes that are potentially cell cycle-related, we clustered expression data for a pool of 2000 genes in *S. pombe *having highly consistent cell cycle phase characteristics (see Additional file 8 for the list of the 2000 genes). As described in the Results section, the depth of the pool enabled us to detect new, unexplored modes of transcriptional and post-transcriptional gene regulation in *S. pombe*.

In contrast with previous studies [26], the larger pool size in the present study posed a computational challenge to conventional clustering, which was compounded by the relatively large number of time course and non-time course regulatory experiments. Without the regulatory signatures, solely time course clustering of such a large number of genes produced noisy clusters (data not shown). However, common clustering algorithms that do not distinguish between heterogeneous types of data are more likely to identify primarily the genes with the most consistent periodic profiles across experiments, while identifying the remaining majority of genes as "noisy" and clustering them arbitrarily. To address this, we enhanced the power of detection of an earlier Bayesian co-clustering algorithm [15] with the capacity to produce clusters of genes that are co-expressed in many, but not necessarily all of the experiments. Using a mixture of regressions based on the cluster-experiment error variances (see Methods and Additional file 5 Figure S3), the strategy of formalizing the notion of a clustering consensus among independent experiments made our algorithm robust against inter-study variation. As a result, unlike earlier studies that did not use high-throughput data and focused on small-scale networks of biochemical interactions in the *S. pombe *cell cycle ([27,28], our approach based on the comprehensive parts-list offers both broad and specific insights.

An ideal window for exploring an interesting phase-specific sub-network is the early M phase, which is the onset of intense regulatory activity involved in mitosis. The regulation by multiple forkhead TFs of different pathways leading to mitosis is well-studied [2,29-31]. Indeed in our NCA, *fkh2 *displays strong late G2 activity (Figure 1). Recently, Nachman and Regev [10], using a Biochemical Regulatory Network Inference (BRNI) approach, have shown that cell-division specific genes *ace2 *and *fkh2 *act together in a combinatorial regulation way and that *fkh2 and sep1 *are involved in a negative feedback loop that may control regulatory activity at the G2/M phase of the fission yeast cell cycle. Interestingly, *fkh2 *also shows high coordination with *SPBC19G7.04 *(Figure 1 and Additional file 5 Figure S4), a HMG box TF that is periodically expressed (*P *< 10^-33^) at the onset of M phase [9,32]. Assuming that TFs with similar TFA profiles might function together [12], it is interesting to check for possible regulatory associations among these factors targeting the M phase-specific clusters. For instance, a large number of the promoters for genes in cluster 4 (an early mitotic cluster), contained two strong motifs (TGTTTTAC and TGTAWGC) with best matches for binding sites of the forkhead domain (*FoxF2*, *P *< 10^-10^) and HMG box (*HMG-1*, *P *< 0.001) respectively. Many of these promoters actually contain both types of binding sites, and in opposing-strand orientation, potentially indicative of combinatorial regulation (Additional file 5 Figure S5). While HMG-box TFs are known to associate with forkhead TFs for ribosome biogenesis in *S. cerevisiae *[33], their dual role with respect to chromatin, which is partly structural and partly regulatory, might be particularly favorable for the intensely active regulation at the onset of mitosis. For instance, besides acting as a conventional TF, some HMG-box TFs can also induce changes in the DNA structure that enhance binding of other TFs [34]. On the other hand, a forkhead TF that binds to condensed chromatin during mitosis could use chromatin-remodeling for regulation [35]. In *S. cerevisiae*, *fkh2 *not only regulates the G2/M specific *clb2 *(cyclin B) cluster [36], it also represses *clb2 *with the help of a chromatin-remodeling ATPase that re-positions nucleosomes in the *clb2 *promoter [37].

Intriguingly, no systematic study of HMG-box TFs in fission yeast is known although it is the only TF family that has (60%) more regulatory members present in *S. pombe *than in *S. cerevisiae *[11]. As a regulatory hub in our predicted early M phase network, *SPBC19G7.04 *is linked with well-known periodically expressed mitosis and cell division proteins such as *klp6 *and *klp8 *(kinesin microtubule motor proteins required for chromosome segregation), some of which also contained both forkhead and HMG-1 motifs in their upstream regulatory sequences (data not shown) and are potential candidates of associated regulation.

While pointing out the gap in *S. pombe *regulatory cascade as compared to *S. cerevisiae*, it was suggested that post-transcriptional regulation might play a major role in the much longer G2 phase of *S. pombe *[8]. With the help of our large gene pool, we focused on two early-to-mid G2 clusters (25 and 26) containing weakly expressed ribosome biogenesis genes. Ribosome biogenesis possibly involves both transcriptional and post-transcriptional steps of gene expression regulation [38], with diverse control elements in budding and fission yeasts [5]. In *S. cerevisiae*, genes encoding factors involved in ribosomal RNA (rRNA) synthesis and ribosome assembly were among those having the least stable transcripts [39]. Given that orthologous genes could have similar turnover across species [40], it is possible that mRNA stability plays an important, if not critical, role in the multi-layered regulation of ribosome biogenesis genes in *S. pombe *[23].

Transcript stability is often regulated by specific interactions between *cis*-elements in the 3' untranslated region (UTR) of mRNAs and hundreds of different RNA binding proteins (RBPs) in the cell [41,42]. Using some of the recently developed resources which specifically search for RNA motifs that may be recognized by different RBPs [17,43,44], we identified highly conserved sequence and structure based RNA motifs in the 3' UTR sequences of 65 ribosome biogenesis genes. Interestingly, these RNA motifs were not significant in the *S. cerevisiae *orthologs of the 65 genes (the only 3' UTR motif reported for nucleolar proteins in the catalog by [43] is GAA.UAUUCA, a distinct motif). Neither were the motifs significant in other gene clusters in *S. pombe *such as the M phase cluster 31. It is therefore possible that the motifs we identified are bound by proteins that perform highly distinctive (species-, location- and phase-specific) post-transcriptional regulation.

Statistical analysis of the time course profiles of the 65 ribosome biogenesis genes containing the motifs indicated low RNA turnover. Despite the general pattern of their expression -- peaking early in G2 followed by fast decay by mid G2, there were exceptions (Figure 6B and [20]) -- and hence the reported motifs may not have a uniformly destabilizing effect. Therefore, we suspect that with more detailed experiments, further regulatory classification is possible. For instance, several genes containing the RNA motifs (such as *lcp5, rrp5, rrp9, utp14, esf2, utp6, utp16*) encode small nucleolar U3 RNP (ribonucleoprotein) associated proteins that form parts of a complex involved in rRNA processing and ribosome biogenesis [45]. Indeed post-transcriptional regulation of such a specific functional class has been observed recently [46]. Thus, it is possible that motifs such as those reported above could act as control elements not only for regulating the level, but also the quality of RNA as needed for ribosome biogenesis and other processes in *S. pombe*.

Finally, we understand that like any computational derivation, our gene and module networks are based on modeling assumptions which may not fully capture the complexity of the multi-layered regulatory program of the *S. pombe *cell cycle. While they are based on different types of data from many experiments, the networks could still be enhanced by future studies from various "omic" approaches. For instance, detailed ChIP-on-chip studies, which have greatly enhanced our understanding of the local protein-DNA interactions in *S. cerevisiae*, could provide similar support to our networks.

## Conclusion

Our fission yeast regulatory network can form the starting point for a variety of inquires. Together with different supporting genome-wide data for the organism such as its sequence [47], proteome [21], localization [48], expression intermediates [23], interactome [49], orthology [50], etc., it offers the scope for a wide range of analysis. In particular, as shown above, the underlying parts-list can be mined for insights on different phase-specific regulatory mechanisms involved in the cell cycle. It also provides a basis for generation of plausible hypotheses for experimental investigation. In this direction, we provided new testable evidence for the hypothesis [8] of post-transcriptional regulation of the G2 phase in *S. pombe *in the form of new RNA motifs for the ribosome biogenesis genes. As future work, we are interested to pursue some of the above results experimentally.

## Methods

### Data

The ten time course microarray experiments on fission yeast cell cycle used in the present study were based on two synchronization methods -- elutriation (Elu) and Cdc25 block-release (Cdc25) -- and referred to as Peng Cdc25 and Peng Elu; Oliva Elut1 & 2 and Oliva Cdc25; Rustici Cdc25 1 & 2 and Elu 1, 2 & 3. See the previously published work [6-8] for more details. The ten regulatory knockout, overexpression or stress experiments, referred to as *Sep1, Sep1p, Nuc2*, N-starvation, *Cdc25, Cdc22, Cdc10, Cdc10*-4hr, *Ace2, Ace2p*, are also previously described [6,8]. The data were normalized by the original experimenters. Therefore, no normalization was performed in this investigation. However, missing values were imputed using the kNNImpute algorithm with the default parameter settings [51]. For post-transcriptional regulation analysis, we used high-throughput proteomic and transcriptomic data for *S. pombe *from [21], and also the mRNA stability study by [23]. Data on ribosomal occupancy and Codon Adaptation Index were also obtained from [23]. Sequence data for fission yeast *Schizosaccharomyces japonicus *was obtained from the Fungal Genomes website of Broad Institute of MIT and Harvard University.

### Data analysis strategy

Our data analysis strategy to identify components in the fission yeast regulatory network consists of two parallel workflows. The first is Bayesian co-clustering [15] of the cell cycle and the regulatory gene expression data sets for 2000 periodic genes (see Additional file 5) followed by circular-circular regression (CCR) [16]. For the median profile of each cluster, a random periods model (RPM) [52] was fit. CCR methodology was applied on the estimated phases of all the median profiles across ten experiments to determine the phase coherence of the clusters over the experiments. The details of the clustering algorithm are fully described in Additional file 5. The second workflow used estimated period parameters from the RPM to obtain significant TF-gene pairwise time lagged correlations as priors for Network Component Analysis (NCA) [12]. Using Peng Cdc25 and Peng Elu data separately, we inferred significant TF activities (TFAs) of 36 TFs during the cell cycle and identified their potential targets (see Additional files 2 and 9 respectively). The activity of a TF is determined by the effect (suitable log-linear decomposition of the gene expression matrix according to the proposed network connectivity) of the TF on its downstream targets as a function of time. The Gene Regulatory Network Inference (GRNInfer) software [53] with default parameter settings (*λ *= 0.0 and threshold = 1 × 10^-3 ^controlled the sparseness and the complexity of the network respectively) was used to reconstruct the "consistent" interactions of the 36 TFs with significant activities and the 531 regulatory targets of the TFs based on the five smaller Rustici time course experiments (Elu 1&2 and Cdc25 1, 2&3). The contribution of each experiment to the reconstruction of the gene network was weighted by the average signal to noise ratio from the RPM (Additional file 5 Table S1). Consistent interactions are the connections of network nodes that are reproduced reliably in each of the five experiments (see Additional file 3 for the network). Listed below are the details of the models, algorithms and statistics used for the correlation analysis.

### Bayesian co-clustering methodology

In this section, we are not introducing new methodology but pointing out extensions to an earlier algorithm described elsewhere [15,54]. We enhanced the algorithm with strategies to co-cluster data from different types of high-throughput experiments. This was achieved by using suitable basis functions to model the individual data types. In particular, we combined time course cell cycle expression data with TF mutant data to identify co-regulated modules. Further, we used a mixture of regressions based on the cluster-experiment error variances to formalize clustering consensus among independent experiments. This allowed the algorithm to produce clusters of genes that are co-expressed in many, but not necessarily all of the experiments thus increasing statistical power and robustness against inter-study variation. For the sake of completeness and reproducibility, we described the methodological details and the steps of the algorithm in Additional file 5.

### TF-gene pairwise time-lagged correlation analysis

We started with a curated list of 125 known transcription factors in *S. pombe *with identified protein domains [11]. Each TF expression time course was transformed with a sigmoid function:

(1)

where *x*_*i *_is relative expression at the *i*^*th *^time point for a given TF,  is the mean of the TF expression profile over all time points and *s s*is the standard deviation [55] assuming that the transformation sufficiently models the activation of a gene by a TF in a nonlinear (sigmodial) fashion.

The time lag correlation between the *j*^*th *^TF's and *g*^*th *^gene's time course expression profiles over the first full time period of the cell cycle experiment (which is least affected by loss of phase synchronization, and allows a lag between the activation of the TF and the peak phase of the gene) was computed using Spearman's rank correlation. Let *φ*_*g *_denote the estimated phase angle of gene and let *T *(in minutes) denote the estimated cell cycle period of a particular experiment [52]. Then the phase angle for a gene or TF in minutes is given by . The TF-gene specific time lag (phase separation) is computed as

(2)

For *j*^*th *^TF with intensity values *f*(*x*_*i*_), *i *= 1,2,..., *m *+ 1, and a gene *g *with expression values *y*_*g *_= (*y*_*g*1_, *y*_*g*2_,..., *y*_*gN*_) the most significant Spearman's rank correlation coefficient *r*_*jg *_is determined between *f*(*x*_*i*_), *i *= 1,2,..., *m *+ 1 and the sub-vector of the full time course of the gene *g *, where *m *is index of the time point corresponding to the first full period of the cell-cycle for the *g*^th ^gene over values of the offset *k*, an integer in the "inclusive" range {-3,...3}, to account for uncertainty in the determination of the true lag. The significance of each *r*_*jg *_is determined with a two-sided *p*-value based on 100,000 random permutations corresponding to the null hypothesis that the TF-gene pair is uncorrelated.

Further criteria were applied to filter out spurious and non-significant TF-gene correlations. First, genes with known peak expression at phase transition points in the cell cycle in *S. pombe *(such as protein-serine/threonine kinase *hsk1 *required for S phase initiation) were used as demarcations to assess the phase separation between the expression of a TF and the response of a gene. A pair is filtered if its phase separation interval contained demarcation genes from multiple transition points or exceeded 120 degrees. If so, the p-value of that correlation was assigned to 1. This phase-specificity imposed a biological constraint on the TF-gene pairs, limiting the extent of a TF's regulatory influence based on its phase information. Second, within each of the ten experiments the p-value for every pair was subjected to multiple hypotheses testing with a q-value threshold of 0.05. Only a TF-gene pair with significant correlations (each of *q *< 0.05, and having the same sign) in at least a third of the nine experiments was qualified for NCA based on data from the tenth. Assuming a Binomial distribution model, the probability that a TF-gene correlation was significant in k ≥ 3 of the n = 9 experiments by chance alone is 0.0023. The Peng Cdc25 and Elu data sets were individually held out of the correlation analysis and reserved for separate runs of NCA.

Phase Coherence among multiple experiments

In this section we are not introducing any new methodology but describing previously published work [16] for completeness sake for reader's convenience so that the paper is self contained. For any given experiment, the phase angle corresponding to cell cycle genes are points on a circle which intrinsically satisfy an (un)known order amongst themselves due to their biological functions. However, due to variability in the underlying data, it is possible that the order of the estimated phase angles of the cell cycle genes may not be same across experiments. If order of the phase angles are preserved across a pair of experiments, then clearly one can align the phase angles of the two experiments by simply rotating the one of the circles and then moving the points within the circle until the two circles are as close to each other as possible. This can be accomplished using circular regression [56]. If there are *k *cell cycle experiments then one can perform *k *pairs of circular regressions to align all the circles. Using this principle a methodology was developed [16] with which we could evaluate if a given collection of gene clusters shows phase coherence in multiple independent cell-cycle experiments. It is said to be coherent if the ordering of the estimated median phase angles of the clusters across experiments is preserved up to circle-circle regressions. That is, by performing a series of circle-circle regressions, it should be possible to "align" the clusters' phases. A p-value was derived for performing the test using a previously described method [16]. In our analysis of the median values from the clusters of the time course data, one cluster was not represented in the two Peng experiments and therefore was excluded from CCR.

### Network Component Analysis and other programs

To infer transcription factor activities (TFAs), we used the Network Component Analysis (NCA) program [12]. The activity of a TF is the effect (suitable linear combination of the gene expression according to the proposed network connectivity) of the regulator on the downstream targets it controls at a given time point. Since the NCA algorithm allows prior knowledge of the regulation between the *i*^th ^gene and *j*^th ^TF to be input as a connectivity matrix, we used only the significant TF-gene correlations from the time lag correlation analysis for this purpose and as follows:

(3)

where +1 is activation, -1 is inhibition and 0 no interaction. The Peng Cdc25 and Elu time course experiments were individually used as the gene expression data input to NCA. Briefly, NCA models gene expression as a function of TFA and the corresponding control strengths (CS) of a transcription factor target interaction. In log-log form the model is written in matrix notation as:

(4)

where E_*ij *_= log(*g*_*i*_(*t*_*j*_)/*g*_*i*_(*t*_*o*_), *g*_*i*_(*t*_*j*_) is the expression of *i*-th gene evaluated at time *t*_*j*_, A is the regulatory network of control strengths where A_*ij *_denotes the control strength of transcription factor *j *on gene *i *(A_*ij *_= CS_*ij*_), P_*ij *_= log(TFA_*i *_(*t*_*j*_)/TFA_*i *_(*t*_0_)) with TFA_*i *_(*t*_*j*_) being the *i*-th transcription factor activity evaluated at time *t*_*j*_, and Γ represents the noise from the DNA microarray experiment. The dimensions of E, A and P are (N × M), (N × L) and (L × M), respectively, where N is the number of genes in the network, M is the number of data points or experiments conducted, and L is the number of transcription factors used in the analysis. TFA_*i *_(*t*_*j*_) and CS_*ij *_are estimated using the regulation (indicator) matrix (equation 3), the gene expression data and the Expectation-Maximization (EM) algorithm with an epsilon = 1 × 10^-6 ^for conversion. Using the Peng Cdc25 data 39 TFs with TFAs on 784 were obtained and using the Peng Elu data 47 TFs with TFAs on 894 targets were obtained. The intersection of the results from the two NCAs led to the identification of 36 TFs with TFAs (Additional files 2 and 9) on 531 regulatory targets.

The R package CircStats was used to compute circular statistics of the peak phases of genes in every cluster. By coupling gene-wise phase information for 10 experiments with the meta-analyzed list of all genes it was observed that approximately 1,900 genes could be rejected (after FDR adjustment) at significance level 0.05 for the null hypothesis that a gene's phases are uniformly distributed across 10 experiments. The same count increased to more than 2,100 genes when the experiment in which a gene deviated most from its median phase was excluded [57]. Hence we chose the top 2,000 genes in the above list (we computed the circular variance of their phases) for our clustering purposes (for gene list see Additional file 8). A p-value threshold of 0.01 was used for the test of circular uniformity to identify diffuse clusters.

For binding site analysis, BioProspector [58] was used to discover conserved DNA motifs in the upstream regulatory regions of co-expressed clusters. Only the two most significant motifs per cluster, both with p-value less than 10^-9^, were output. No motif was output for a diffuse cluster. A motif clustering program was used to filter the redundant motifs [59] as well as to recognize the previously known motifs [6,8]. The following databases were used: protein-protein interactions obtained from BioGRID [49], p-values for periodicity and regulation of expression profiles from Cyclebase.org [32], candidate binding sites from JASPAR [60], and upstream sequences from GeneDB [61]. The DNA motifs were searched against JASPAR with STAMP [62] and plotted with MotifViz [63]. The RNA motifs were identified with FIRE and RNApromo [17,44]. Sample skew and sample excess kurtosis of time courses were computed with the R package FinTS. The skew was computed for the first full range of G2 phase in Peng Cdc25 time courses spanning 150-270 min., and the kurtosis for the sub-range 190-270 min. to focus on the right tail. Data on AUG Context Adaptation Index and ribosome occupancy were obtained from [23]. The p-value of gene expression (Table 3) is due to the gene-specific P(reg) entry in Cyclebase.org [32]. The periodicity and dominant Fourier frequency for the log_10_(TFA) profiles were determined with Fisher's *g*-statistic (Table 1) and the Average Periodogram (Figure 2) using GeneCycle [13]. The module network (Additional file 4) was computed using four Cdc25 and four Elu data (Oliva Elu 1&2 not used) with Genomica [64]. Gene Ontology based associations were computed with Genecodis [65].

## List of abbreviations

Cdc25: Cdc25 block-release; CCR: circle-circle regression; CS: control strength; EM: expectation-maximization; Elu: elutriation; GO: Gene Ontology; TF: transcription factor; TFA: transcription factor activity; NCA: network component analysis; GRNInfer: Gene Regulatory Network Inference; rRNA: ribosomal RNA; UTR: untranslated region; RNP: nucleolar ribonucleoprotein; RBPs: RNA binding proteins; RPM: random periods model.

## Competing interests

The authors declare that they have no competing interests.

## Authors' contributions

PRB contributed to the analysis of the time course data to generate TF-Gene correlations, TFAs, circle-circle regression and participated in writing the manuscript. NAH performed the Bayesian clustering. RG performed the motif analyses. LL performed the Gene Ontology analysis. SDP fitted random periods models to estimate phase angles and provided helpful suggestions with the statistical analyses. SP conceived the research project, designed algorithmic strategies and wrote the manuscript. All authors read and approved the final manuscript.

## Supplementary Material

Additional file 1 **Regulatory targets of the 36 TFs**. Regulatory targets of the 36 TFs. Each target is denoted as to whether it was found to be regulated by the TF using NCA and either one or both of the Peng gene expression time course data sets. Click here for file

Additional file 2 **TFA for 36 TFs based on Peng Cdc25 gene expression time course data**. The NCA generated TFAs for the 36 TFs using the Peng Cdc25 gene expression time course data. Click here for file

Additional file 3 **Gene regulatory network for *S. pombe *generated from Rustici Cdc25 and Elu gene expression time course data**. A directed acyclyic graph (DAG) dot text file containing the interactions between regulatory targets. The network can be opened and viewed with the GVedit function of Graphviz. Click here for file

Additional file 4 **M and G1 phase regulatory modules**. An XML file containing the *S. pombe *M and G1 cell cycle phase regulatory modules. The data can be opened and viewed with the GeneXPress software. Click here for file

Additional file 5 **Supplemental materials**. Supplemental materials containing the Bayesian co-clustering algorithm and supplemental figures and tables. Click here for file

Additional file 6 **Cluster assignments**. The assignment of each of the top 2000 *S. pombe *genes (those with highly consistent cell cycle phase characteristics) to a cluster using the Bayesian co-clustering algorithm. Click here for file

Additional file 7 **Likelihood scores for the occurrence of RNA motifs in the 65 co-regulated genes from clusters #25 and #26**. The likelihood scores for the occurrence of RNA motifs in the 65 co-regulated genes from clusters #25 and #26. Click here for file

Additional file 8 **The top 2000 *S. pombe *genes with highly consistent cell cycle phase characteristics**. The top 2000 *S. pombe *genes with the lowest circular variance. Click here for file

Additional file 9 **TFA for 36 TFs based on Peng Elu gene expression time course data**. The NCA generated TFA for the 36 TFs using the Peng Elu gene expression time course data. Click here for file
