# Supplementary material for: Dissecting the fission yeast regulatory network reveals phase-specific control elements of its cell cycle
Source: BMC Syst Biol. 2009 Sep 16;3:93. doi: 10.1186/1752-0509-3-93 (PMC2758837; doi:10.1186/1752-0509-3-93)
Supplement: Additional file 5 — Supplemental materials. Supplemental materials containing the Bayesian co-clustering algorithm and supplemental figures and tables. [file 1752-0509-3-93-S5.pdf]

Supplemental Materials:

Dissecting the Fission Yeast Regulatory Network Reveals Phase-Specific Control Elements of its Cell Cycle

Pierre R. Bushel[1,5,#,*], Nicholas A. Heard[2,#], Roee Gutman[3], Liwen Liu[1], Shyamal D. Peddada[1], Saumyadipta Pyne[4,*]

[1]Biostatistics Branch, National Institute of Environmental Health Sciences, Research Triangle Park, NC 27709 USA

[2]Department of Mathematics, Imperial College, London, UK

[3]Department of Statistics, Harvard University, Cambridge, MA 02138, USA

[4]Broad Institute of the Massachusetts Institute of Technology and Harvard University, 7 Cambridge Center, Cambridge, MA 02142, USA

[5]Department of Statistics and Department of Environmental Health, Harvard School of Public Health, Harvard University, Cambridge, MA 02115, USA

[#] These authors have contributed equally to this work

*Corresponding authors: spyne@broad.mit.edu, bushel@niehs.nih.gov

Bayesian Co-clustering Methodology

1. The Co-clustering Regression Model

Let $f_{ge}(t), t \geq 0$, denote the expected value of the relative expression level $y_{ge}(t)$, at time *t* for an individual gene *g* in experiment *e*. Assuming that the random errors $\varepsilon_{ge}(t)$ are standard normally distributed, we model the expression value $y_{ge}(t)$ using the regression model $y_{ge}(t) = f_{ge}(t) + \sigma_{ge}\varepsilon_{ge}(t)$. Following the methodology of [1, 2], we represent $f_{ge}(t)$ by a linear combination of a set of basis functions, specified for each experiment type. To model periodic profiles, we depart from the piecewise polynomial representation of the earlier versions and instead consider a Fourier representation. Thus we have

$$f_{ge}(t) = \alpha_{ge} + \beta_{ge,0}t + \sum_{j \geq 1} \beta_{ge,j} X_j(t), \qquad (1)$$

with basis functions given by

$$X_j(t) = \begin{cases} \cos((j+1)t/2) & j \text{ odd} \\ \sin(jt/2) & j \text{ even} \end{cases}$$

For non-time course data, we have $f_{ge}(t) = \alpha_{ge}$. To identify co-clustered genes, it is reasonable to use the first full cycle time period data for every gene since it is least affected by the loss of cell phase synchronization. Clearly, using asynchronous data produces noisy clusters. Moreover, if we restrict ourselves to the first period of the cell cycle, then it is easy to see that the RPM model of [3] can be approximated by the above model (1). An advantage of using (1) is that it is linear in the unknown parameters and hence is easy to fit. Thus, we can reasonable assume that the model is equivalent to the RPM model.

2. Combined cluster model

The model-based clustering strategy assumes that for each experiment $e \in \{1,2,...,E\}$, genes in cluster k share the same regression function $f_{ge} = f_e^{(k)}$ and a common error variance $\sigma_{ge}^2 = \sigma_e^{2(k)}$. For regression coefficients we use the Gaussian prior and for error variances we use the inverse gamma prior, which are commonly used conjugate priors [1]. The model specification is completed with a prior distribution placed on the clustering configuration C. Here we use a uniform prior over the space of all clustering configurations. To avoid outlier genes from forming stray clusters, we limited clusters to a minimum size of five genes.

The clustering algorithm of [4] requires calculations involving $V^* = (X'X + \upsilon^{-1}I)^{-1}$, the posterior covariance matrix of the basis function coefficients for each cluster. Here $X(t)$ is a matrix whose columns are the basis functions described above and I is the identity matrix. Since we have a relatively large number of experiments, including some long time courses, these matrix inversions and subsequent matrix multiplications are computationally demanding. Gram-Schmidt orthonormalization of the basis functions is therefore used to reduce the computational burden, without changing the basic structure of the regression model as in [4].

3. Automatic relevancy detection for experiments within a cluster

Since data used in this study are from independent experiments performed under varied conditions, there could be considerable variation among the expression profiles. Consequently, even though a pair of genes is theoretically co-expressed, often the observed data from the ten experiments may reveal that they are co-expressed in only a subset of the experiments and not necessarily in *all* ten experiments. Thus one of our goals is to identify subsets of co-expressed genes even though, due to variations among the observed data, they may not necessarily be co-expressed in all ten experiments.

The above problem can be formulated within the Bayesian framework by using a mixture distribution for the error variance $\sigma_e^{2(k)}$. If an experiment does not demonstrate co-expression of the genes in cluster *k*, then the corresponding data suggest a regression model with a relatively high variance. We consider a two component mixture model, with both components modeled by inverse-gamma distribution $\mathrm{I}\Gamma(\gamma, \lambda)$, the conjugate prior. Hence we propose the following model

$$\sigma_e^2 \sim w_e \mathrm{I}\Gamma(\gamma, \lambda) + (1 - w_e)\mathrm{I}\Gamma(\gamma', \lambda')$$

where $(\gamma, \lambda), (\gamma', \lambda')$ are chosen to reflect low and high variance, respectively. In our application we chose $\gamma = \gamma' = 1$, and $\lambda = 0.5, \lambda' = 10$. The resulting probability density functions for the two components are shown in Figure S5.

Let $z_e^{(k)}$ be a latent, unobserved variable (similar to the unknown clustering) which determines whether $\sigma_e^{2(k)}$ is drawn from the first or second component of the mixture, with probabilities w_e and $1-w_e$ respectively. Thus our model space and the search space are extended to finding the optimal clustering and component allocations (C, z) where z is a vector comprised of the component allocations $\{z_e^{(k)}\}$ for experiment e and cluster k in C. Based on prior assumptions [5] about the relative influences of regulatory and cell cycle experiments, for symmetry we set w_e equal to 0.8 and 0.2 respectively for these two types.

4. Clustering Algorithm

Given the observed data, the above probabilistic model results in a posterior distribution on the clustering and variance component allocations, given up to proportionality by,

$$\pi(C, z) = p(C)p(z \mid C)p(y \mid C, z), \qquad (2)$$

where the final term in the product is the density of all of the observed data given (C, z). We then use π as the objective function for our agglomerative clustering procedure.

5. Agglomerative clustering

Although the statistical model described here is an extension of [1, 4], the agglomerative clustering algorithm of [4] can still be applied here. Higher potential scores π can be achieved through various schemes to relocate genes to different clusters after agglomeration. One such method is used here, whilst full details of a range of methods will appear in a separate paper. The revised agglomerative clustering algorithm for N genes proceeds as follows:

- *Step 1:* Start with C=N clusters, each cluster containing the expression levels for one gene. Calculate the potential π_N using (2).
- *Step 2:* Let (C, z) be the current configuration. For each pair of clusters $k, l \in C$, let $C^{(k,l)}$ represent the hypothetical clustering resulting from their merger and $z^{(k,l)}$, the corresponding variance component allocations which maximize $\pi(C^{(k,l)}, z^{(k,l)})$. Calculate

$$c_{kl} = c_{lk} = \frac{\pi(C^{(k,l)}, z^{(k,l)})}{\pi(C, z)} \qquad (3)$$

- *Step 3:* For each cluster k, identify the closest other cluster according to the metric in (3) and the corresponding maximum closeness value

$$k' = \arg\max_{l} \; c_{kl} \;, \qquad c_k = c_{kk'} \;.$$

- *Step 4:* Find the cluster $\hat{k}$ with largest c_k value, and merge with cluster $\hat{k}'$ to form a new cluster $\hat{k}$. Set C=C−1 and re-label the other remaining clusters accordingly. Let $\Delta = \{\hat{k}\}$.
- *Step 5:* Following this merger, find the gene whose transfer into a different cluster now causes the biggest increase in π. Move the gene to this higher probability cluster. If the two clusters involved were ℓ and ℓ', let $\Delta = \Delta \cup \{\ell, \ell'\}$.
- *Step 6:* Repeat *Step 5* until no gene can be transferred to a different cluster and still cause an increase to π.
- *Step 7:* Calculate the revised posterior kernel value π_C for the current configuration.

- *Step 8:* Take each cluster $k \in \Delta$, and for each $l \in \Delta$ calculate the closeness to cluster k, $c_{k\ell} = c_{\ell k}$, and hence identify the new nearest cluster to k, $\hat{k}'$.
- *Step 9:* Repeat *Steps 3-8* until C=1.
- *Step 10:* Looking back over the clusterings visited, find the number of clusters C in the hierarchy maximizing the posterior distribution, $\text{argmax}_C \, \pi_C$. This is our optimal configuration (C^*,z^*).

Note that Step 9 determines the number of clusters in our optimal clustering. To avoid small clusters which may be difficult to interpret, we limited this analysis to clusters containing at least 5 genes.

Supplementary Figures and Tables

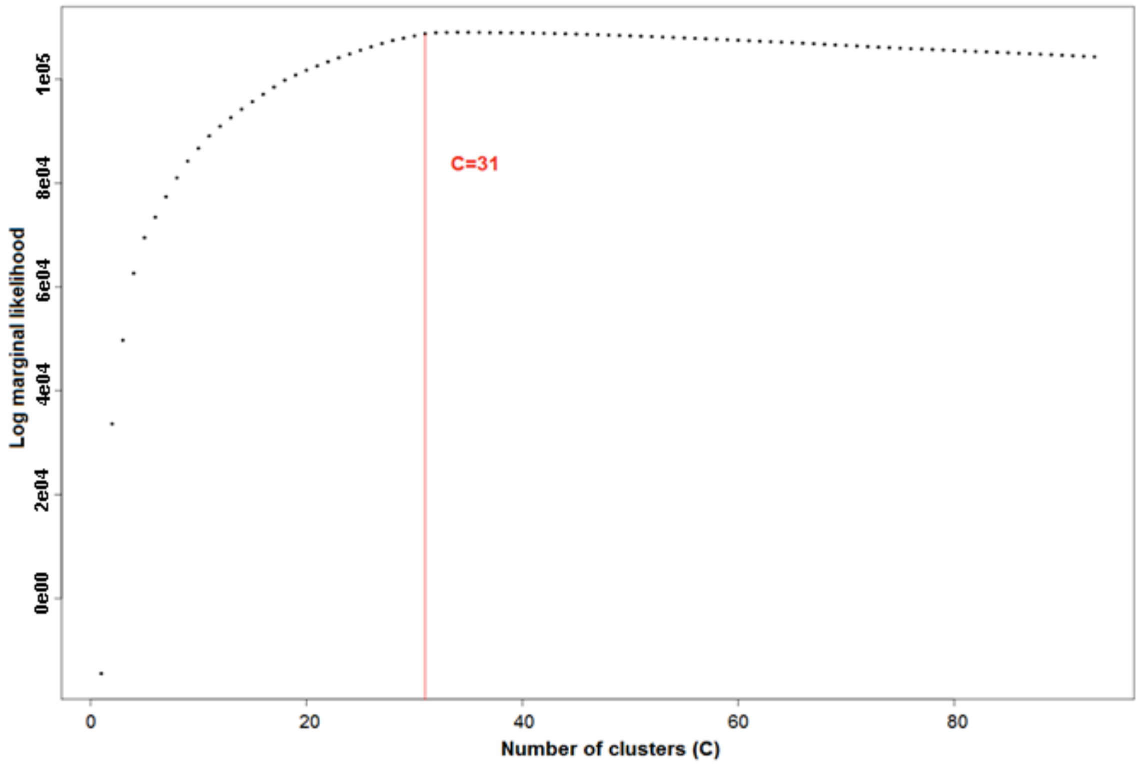

Figure S1. **Determination of optimal number of clusters.** The log (marginal) likelihood score achieved by the clustering algorithm is plotted against the number of clusters. The red line represents the optimal number of clusters (C=31).

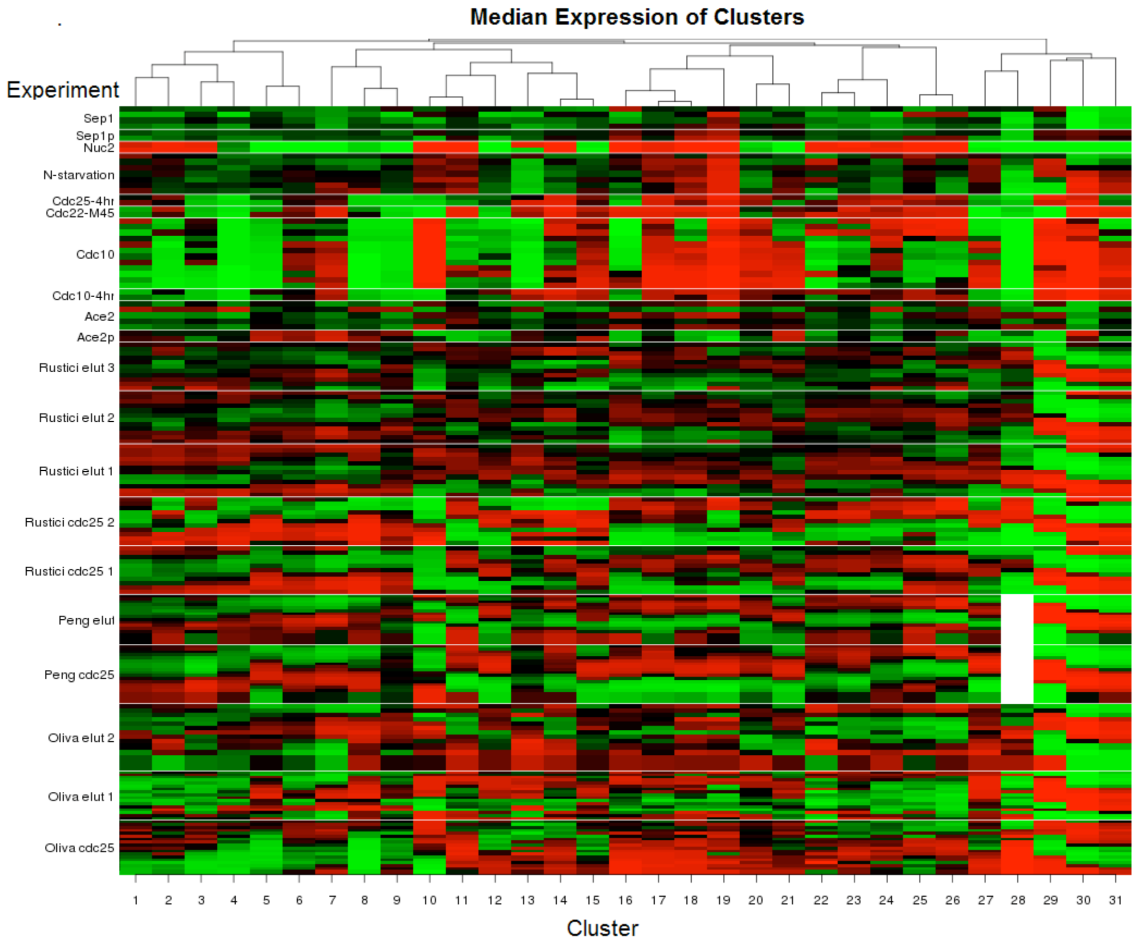

Figure S2. **Overall regulatory signature of every cluster.** For every regulatory and time course experiment, median expression of all genes within each of the 31 clusters is plotted.

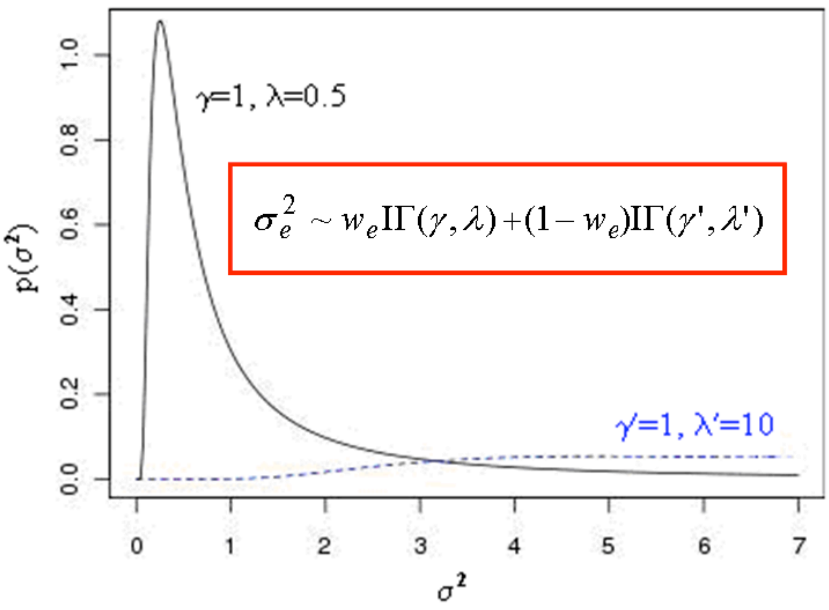

Figure S3. **Formalizing a relaxed consensus-building strategy.** A two component mixture model of experiment-wise error variances allows an experiment to agree/disagree with a co-expressed clustering result based on low/high variance.

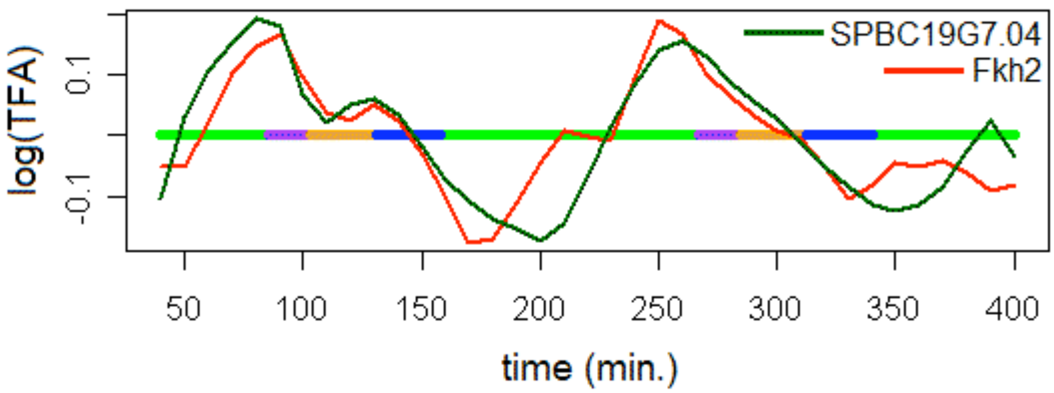

Figure S4. **Inferred transcription factor activities.** The curves represent the activities of two transcription factors as $\log_{10}$(TFA) values computed with Network Component Analysis (NCA) of Peng Cdc25 data. Activities of the TFs Fkh2 and *SPBC19G7.04* peak and trough at different phases of the cell cycle with high correlation. The colored band in the middle represents cell cycle phases: M (purple), G1 (orange), S (blue), G2 (green).

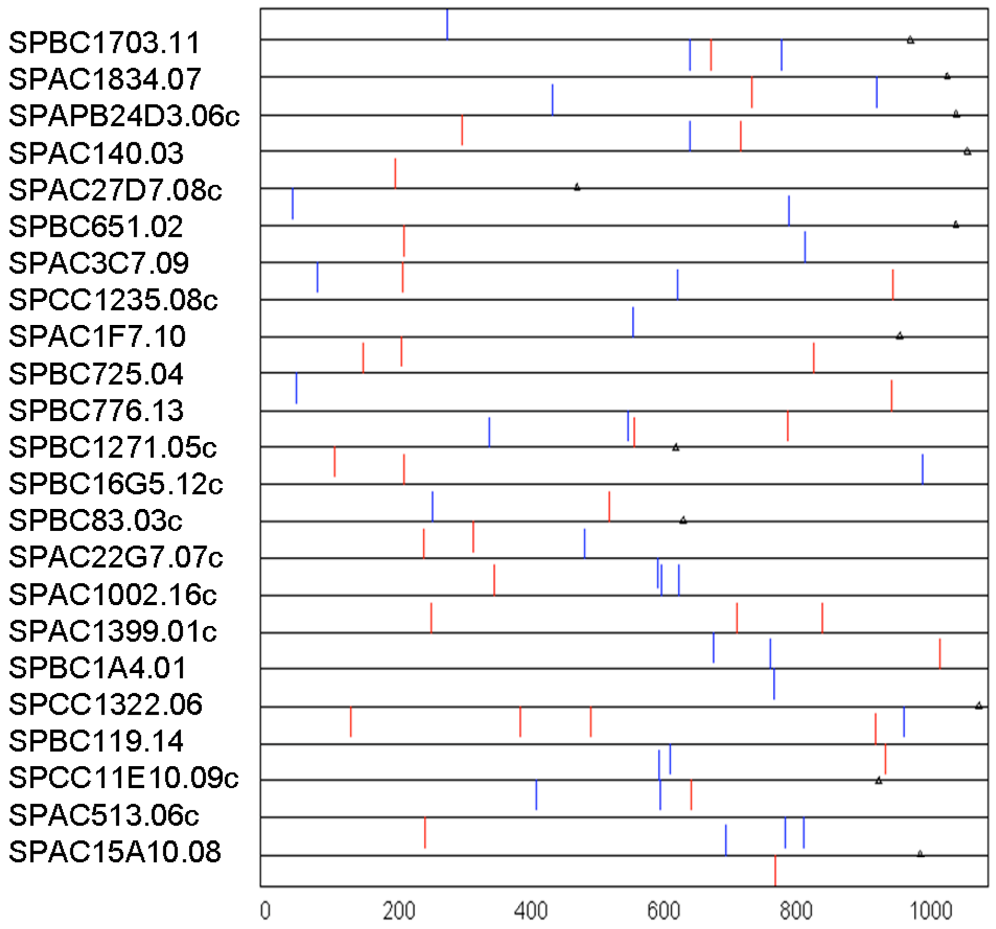

Figure S5. **Putative TF binding sites in the promoter sequences of G2/M genes.** Red and blue spikes represent statistically significant motif matches for potential forkhead and HMG-1 box binding sites in the upstream sequences of genes from Cluster 4 (see Table 2 in main text). A black triangle marks the end of an intergenic region.

Table S1. **Average signal to noise ratio (SNR) across all genes and weights for GRNInfer gene network reconstruction.** SNR is defined as the regression sum of squares divided by the error sum of squares.

Exp	Average SNR	Weight
SNR(Rust cdc 2)	57.60720918	0.220836
SNR(Rust cdc25 1)	64.65589491	0.247857
SNR(Rust Elu 2)	48.37108429	0.185429
SNR(Rust Elu 3)	42.1047688	0.161407
SNR(Rust Elut 1)	48.12120962	0.184471

References

1. Heard NA, Holmes CC, Stephens DA, Hand DJ, Dimopoulos G: **Bayesian coclustering of Anopheles gene expression time series: study of immune defense response to multiple experimental challenges**. *Proceedings of the National Academy of Sciences of the United States of America* 2005, **102**(47):16939-16944.

2. Troyanskaya O, Cantor M, Sherlock G, Brown P, Hastie T, Tibshirani R, Botstein D, Altman RB: **Missing value estimation methods for DNA microarrays**. *Bioinformatics (Oxford, England)* 2001, **17**(6):520-525.

3. Liu D, Umbach DM, Peddada SD, Li L, Crockett PW, Weinberg CR: **A random-periods model for expression of cell-cycle genes**. *Proceedings of the National Academy of Sciences of the United States of America* 2004, **101**(19):7240-7245.

4. Heard NA, Holmes CC, Stephens DA: **A quantitative study of gene regulation involved in the immune response of anopheline mosquitoes: An application of Bayesian hierarchical clustering of curves**. *Journal of the American Statistical Association* 2006, **101**(473):18-29.

5. Oliva A, Rosebrock A, Ferrezuelo F, Pyne S, Chen H, Skiena S, Futcher B, Leatherwood J: **The cell cycle-regulated genes of Schizosaccharomyces pombe**. *PLoS biology* 2005, **3**(7):e225.
